# Quality of life in individuals living with HIV/AIDS attending a public sector antiretroviral service in Cape Town, South Africa

**DOI:** 10.1186/1471-2458-14-676

**Published:** 2014-07-03

**Authors:** Mweete D Nglazi, Sacha J West, Joel A Dave, Naomi S Levitt, Estelle V Lambert

**Affiliations:** 1 MRC/UCT Research Unit for Exercise Science and Sports Medicine, Department of Human Biology, University of Cape Town, Cape Town, South Africa; 2 Department of Sport Management, Human Performance Laboratory, Cape Peninsula University of Technology, Cape Town, South Africa; 3 Division of Diabetic Medicine and Endocrinology, Department of Medicine, University of Cape Town, Cape Town, South Africa; 4 Chronic Disease Initiative for Africa (CDIA), Cape Town, South Africa

**Keywords:** Health related quality of life, HIV or AIDS, Health outcomes, EQ-5D

## Abstract

**Background:**

Health related quality of life (HRQoL) is an important outcome helping to understand the impact of antiretroviral therapy (ART). We examined and compared the HRQoL in relation to ART status among HIV-infected patients in a public sector service in Cape Town, South Africa. In addition, we aimed to examine the relationship between ART status and HRQoL according to CD4 count strata.

**Methods:**

A cross sectional study sample of 903 HIV-infected patients who were categorized as not receiving ART (ART-naïve) or receiving first-line ART for > 6 months (ART). HRQoL outcomes were compared in the two groups. HRQoL was assessed using the EQ-5D (five domains) and Visual Analogue Scale (EQ-5D VAS).

**Results:**

Of the total sample, 435 were categorised as ART naïve (76% women) and 468 were on ART (78% women). There were no significant associations between groups for most of the EQ-5D domains, however ART-naïve experienced a significantly greater problem with mobility than the ART group. Being ART-naïve (adjusted odds ratio (aOR) 3.08 95% confidence interval (CI) 1.63- 7.89) and obese 2.78 (95% CI 1.24- 6.22) were identified as predictors for increased mobility problems in multivariate analysis. In addition, receiving ART (5.61 difference; 95% CI 2.50 - 8.72) and having some source of income (4.76; 95% CI 1.63 -7.89) were identified as predictors for a higher EQ-5D VAS score. When grouped according to CD4 count strata, there were no significant difference between groups for most of the EQ-5D domains, however the ART-naïve group indicated having significantly greater problems under the CD4 count of >500 cells/μL in the anxiety/depression domain (22.4% vs 8.8%, p = 0.018) and significantly lower EQ-5D VAS scores under the CD4 counts of ≤200 cells/μL (median 80 (IQR 60–90) vs 90 (IQR 80–100), p = 0.0003) and 201–350 cells/μL (median 80 (IQR 70–90) vs 90 (80–100), p = 0.0004) compared to ART group.

**Conclusions:**

HRQoL (self-rated health state) was improved with ART use, including those with immunocompromised status, which may be relevant to the public sector ART program in South Africa.

## Background

Human immunodeficiency virus (HIV) is a global epidemic with 35 million people living with HIV/AIDS worldwide, the majority of whom reside in resource-poor countries. By 2012, sub-Saharan Africa accounted for 70% of the universal total, with South Africa home to the largest number of people living with HIV in the world (6.1 million) [1].

The roll out of antiretroviral therapy (ART) has profoundly improved the grave portrayal of this disease in South Africa. The national ART program commenced in 2004 and the latest WHO statistics show that, at the end of 2012, there were an estimated 2 150 881 HIV-infected people receiving ART in South Africa [2]. However, while ART prolongs the lives of HIV-infected individuals [1], it is associated with a variety of metabolic sequelae, including metabolic abnormalities and morphological body changes [3-5], that may adversely affect quality of life with long-term use [6]. There is, therefore, a growing need to understand the impact of ART use and on health-related quality of life (HRQoL).

Numerous studies have examined HRQoL in HIV-infected individuals the impact of ART on HRQoL in HIV infected adults in South Africa [7-12]. These studies have been either cross-sectional or longitudinal designs. A recent cross-sectional study demonstrated a significant association between ART use and improved HRQoL indicators [7]. A second cross-sectional study found an improvement in physical health only [8]. Another study showed less pain and discomfort and fewer problems with self-care, daily activities and general mobility [10]. In addition, longitudinal studies have shown significant improvements in HRQoL during 7 months [9], 12 months [11] and 24 months [12] of follow-up after ART initiation.

There are, however, relatively fewer studies from South Africa that assessed the effect of higher CD4 cell counts on HRQoL [7,13]. Bhargava and colleagues [13] found the HRQoL of patients receiving ART increased significantly with improvement in CD4 count . Igumbor and colleagues [7] found weak but significant associations between CD4 cell counts and HRQoL in a cohort of treatment-naïve patients and those who had received ART for 12 months.

Globally, there has been concern about how ART-related toxicities may adversely affect HRQoL of HIV-infected individuals. Non-nucleotide reverse transcriptase inhibitors such as stavudine (d4T) have been shown to be associated with metabolic complications such as dyslipidemias, lipoatrophy, peripheral neuropathy and lactic acidosis [14-25]. Owing to toxicity concerns, the World Health Organization (WHO) in 2010 recommended the replacement of d4T with tenofovir (TDF) or zidovudine (AZT) -based first-line regimens which have better safety profiles [26,27].

Despite the World Health Organization recommendation to phase out d4T, the national ART programs of South Africa and other developing countries continue to use d4T as part of their first-line ART regimen. By the end of 2011, 1.1 million people were taking d4T regimens globally, the vast majority in resource limited settings in sub-Saharan Africa [28]. Progress in phasing-out d4T has been tampered by the higher cost of the alternative drugs AZT and TDF, uncertainties regarding whom to give priority to for phase out, the existence of stockpiles of d4T in several countries [28] and the failure of major donors to support the complete elimination of d4T [29]. With the lack of full elimination of d4T from first-line regimens in resource-poor countries, there is a need for studies on the impact of d4T-containing first-line ART regimens on HRQoL in order to inform their national ART programs.

Therefore, the objectives of this study were to establish whether there was a difference in the HRQoL in those patients who were not receiving ART compared to those who were on first-line ART (predominantly d4T-containing regimen for longer than 6 months) in public sector treatment program in Cross roads, Cape Town, South Africa. In addition, we aimed to examine the relationship between ART status and HRQoL according to CD4 count strata.

## Methods

### Study setting and population

This study is a cross-sectional secondary analysis of a subset of data collected from a broader study examining the prevalence of metabolic complications of ART in HIV-infected patients, conducted by the Division of Diabetic Medicine and Endocrinology of the University of Cape Town in 2007. The study site was the Crossroads community health clinic in Cape Town which provides HIV care to over 5000 patients. As per national ART guidelines at the time of data collection, adult patients qualified for public sector ART if they had a CD4 count below 200 cells/μL and symptomatic, and/or WHO Stage four AIDS defining illness (World Health Organisation HIV/AIDS Staging System) [30]. The study population consisted of 435 not yet receiving ART (ART-naïve) and 468 on the South African National Department of Health first-line ART regimen (stavudine (d4T) or zidovudine (AZT), lamuvidine (3TC) and efavirenz (EFV) or neviparine (NVP). At the time of data collection, patients in South Africa only qualified for public sector ART if they had a CD4 count below 200 cells/μL and symptomatic, and/or WHO Stage four AIDS defining illness (World Health Organisation HIV/AIDS Staging System) [30].

The study cohort was selected due to the fact that d4T is stll being used as part of first line regimens in South Africa and other resource-limited countries [2,28] and it is, therefore, important to determine whether the drug has a negative impact on HRQoL.

### Sampling technique

The broader study used convenient sampling technique. Patients were conveniently sampled consecutively from the day list at the Crossroad Community Health clinic; and all patients meeting the inclusion criteria were invited to participate in the study. Study recruitment began in February 2007 with a target sample size of 1650 (Note that the main objectives of the original study from which the study emanates were to estimate and compare the prevalence of metabolic complications in three ART regimen- hence to estimate the prevalence of lipodystrophy at 40% (an assumption derived from the literature) within an ART regimen with a precision of 4% a sample size of 557 per ART regimen is needed and to compare the prevalence of lipodystrophy between the three ART regimens using a chi-squared test with 90% power and 5% significant level a sample size of 552 was needed per ART regimen. A total target sample size of 1650 patients (consisting of 550 per ART regimen) was therefore needed to meet the requirements of both objectives). However, only 1035 patients (approximately 95% of patients approached) consented to participate in the broader study.

### Inclusion criteria

To participate in the broader study, subjects were 18 years and older and were required not to have changed their ART in the past six months. Exclusion criteria included a history of diabetes mellitus or impaired glucose tolerance, active TB, active acute opportunistic infections, severe diarrhoea (>6 stools/day), known renal failure, pregnancy, or had received glucocorticoid therapy within the past six months.

### Measurements

This study formed part of a larger study examining the metabolic complications of ART in South African HIV-positive patients where subjects underwent an oral glucose tolerance test (OGTT), blood pressure measurements, lipodystrophy questionnaire and a neuropathy examination [31]. Those data therefore will not be reported in this paper.

### Body Composition Assessment

The following anthropometric measurements were done in all subjects: weight, height, skinfolds (triceps, biceps, subscapular, supra-iliac, abdominal, thigh and calf), circumferences (waist, hip, arm and thigh) and sagittal abdominal diameter (SAD) [32,33]. All measurements were performed by one trained investigator for consistency of data. All measurements were done in duplicate as previously described [31,34,35]. Sagittal abdominal diameter was measured as the distance between the blades of a portable sliding-beam caliper at the level of the iliac crest (L4-5) after a normal expiration with the subject lying in the supine position on a flat, standard, clinic examining bed.

### Questionnaire

A questionnaire was administered face-to-face to all subjects on entry into the study by trained field workers. General and demographic information, measures of socio-economic status, family medical history, clinical conditions and medication, dietary intake, and the use of drugs and toxins (including alcohol and tobacco) was obtained from the questionnaire. A review of subjects’ clinical records was done to provide all information on prior ART regimen, time on ART and CD4 count data.

### Quality of Life

HRQoL was assessed using a validated EQ-5D (five domains) tool and Visual Analogue Scale (EQ-5D VAS) [36-38]. The EQ-5D comprises of two components- a questionnaire and a visual analogue scale-EQ-5D VAS). The questionnaire part is a standardised measure of health status that includes five domains regarding quality of daily life, namely mobility, self-care, usual activities, pain and discomfort, and anxiety and depression. Each domain has three levels: no problems, some problems and severe problems. The EQ-5D VAS is used to assess overall current health. A score is recorded on a vertical, visual analogue scale with endpoints: 0 being “worst imaginable health state” to 100 being “best imaginable health state”.

### Statistical Analysis

Subject characteristics and HRQoL outcomes were tabulated and stratified by ART status. Proportions were compared for categorical variables. Continuous variables were presented as medians and interquartile ranges unless otherwise stated. All continuous variables were not normally distributed (normality tested using Shapiro-Wilk test) except for waist-to-hip ratio. The Wilcoxon-Rank sum test was used to compare medians and Student’s t test to compare means.

The effect of ART status on HRQoL was examined using logistic regression for dichotomous outcome variables (EQ-5D five domains) and linear regression for the continuous outcome variable (EQ-5D VAS score). Each EQ-5D domain were categorised as “no problems” versus “problems” because the number of subjects that reported “severe problems” were very low, and therefore “some and severe problems” were combined to form one “problems” sub-category. The explanatory variable of interest in all models was ART status. Confounding variables were CD4 count, age, gender, education employment housing density, smoking status, ever drank alcohol, previous TB, and BMI category.

The relationship between ART status and HRQoL according to CD4 count strata was examined using Chi-squared test for dichotomous outcome variables (EQ-5D five domains) and one-way analysis of variance by ranks (Kruskal-Wallis test) with alpha/(k(k-1)) adjustments for multiple comparisons for the continuous outcome variable (EQ-5D VAS score). The CD4 count strata were categorized as ≤200 cells/μL, and 201–350 cells/μL, 351–500 cells/μL and >500 cells/μL according to the recent WHO recommendations and national guidelines for commencing ART [30,39,40].

All analyses were performed using STATA 11.1 (StataCorp, College Station, Texas, USA). The level of significance for the one-way analysis of variance by ranks (Kruskal-Wallis test) was set at adjusted P = 0.0009. However, the level of significance for the other tests was set at P <0.05. 95% confidence intervals were also calculated throughout.

### Ethical approval

The study was approved by the Research Ethics Committee of the Faculty of Health Sciences of the University of Cape Town. Prior to participation in the study, risks and procedures were explained to the subjects, all of whom provided informed consent to participate in the study. The data used for the secondary analysis was delinked from the identity of the study participants.

## Results

### Participant characteristics

The sociodemographic and clinical characteristics, as well as body composition of the participants are presented in Table 1. There were a total of 903 participants (435 were ART- naïve and 468 were on ART). Participants in the ART group were significantly older than in the ART-naive group (median 32 years (IQR 28 – 38) years vs median 35 years (IQR 30–42 years; P < 0.0001). Three times as many women as men in both groups participated in the study (78% vs 22% ART, 76% vs 24% ART-naive). Both groups were similar for level of education and employment status. The ART-naive group differed significantly from the ART group in terms of housing density P = 0.002). A higher proportion of patients reported drinking alcohol in the ART group compared to the ART-naïve group (52% vs 43%; p = 0.006), but smoking rates did not differ.

**Table 1:** Subject characteristics for ART-naïve and ART groups

	**Total**	**ART-naïve**	**ART**	**P value**
	**(n = 903)**	**(n = 435)**	**(n = 468)**	
	**N (%)**	**N (%)**	**N (%)**	
**Age, yrs, median (IQR)**	33 (29–40)	32 (28–38)	35 (30–42)	< 0.0001
**Female**	697 (77.2)	331 (76.1)	366 (78.2)	0.450
**Education**				
No education	58 (6.4)	25 (5.8)	33 (7.1)	0.654
≤ Grade 12	820 (90.8)	399 (91.7)	421 (90.0)	
>Grade 12	25 (2.8)	11 (2.5)	14 (3.0)	
**Housing density (persons/room), median (IQR)**	2 (1–3)	2 (1–3)	1.5 (1–3)	0.002
**Employment**				
Full or part time	250 (27.7)	115 (26.4)	135 (28.9)	0.791
Unemployed	648 (71.8)	317 (72.9)	331 (70.3)	
Other	5 (0.6)	3 (0.7)	2 (0.4)	
**Current smoker**	136 (15.1)	75 (17.2)	61(13.0)	0.077
**Ever drank alcohol**	426 (47.2)	226 (52.0)	200 (42.8)	0.006
**CD4 count, cells/μl median (IQR)**	289 (184–455)	224 (153–437)	318 (222–468)	<0.0001
**Previous TB**	379 (49.8)	254 (64.0)	125 (34.3)	< 0.001
**Treatment regimens**				
d4T 3TC EFV	-	-	238 (51.8)	
d4T 3TC NVP	-	-	169 (36.3)	
AZT 3TC EFV	-	-	11 (2.4)	
AZT 3TC NVP	-	-	45 (9.6)	
Other	-	-	2 (0.4)	
**Duration of treatment (months)**				
d4T	-	-	13.0 (8.0-20.0)	
AZT	-	-	12.0 (7.0-18.5)	
**Body composition**				
BMI, kg/m^2^, median (IQR)	25.2 (22.0 -29.6)	24.2 (21.4-29.0)	26.0 (22.9-30.0)	0.0004
Waist-to-hip ratio, mean ± SD	0.85 ± 0.07	0.84 ± 0.07	0.87 ± 0.08	< 0.01
Sagittal height, cm, median (IQR)	19.3 (17.0-22.0)	18.3 (16.5-21.0)	19.8 (17.5-22.5)	<0.0001
Sum of skinfolds, median (IQR)	108.8 (69.5-158.7)	103.8 (64.4-164.1)	111.5 (74.1-153.8)	0.557
Calf skinfolds, median (IQR)	14.6 (8.4 – 21.6)	15.6 (9.3-22.6)	13.4 (7.8-20.5)	0.012

d4T, Stavudine; AZT,.Zidovudine; 3TC, Lamuvidine; EFV, Efavirenz; NVP, Nevirapine.

The ART group had higher CD4 counts and less cases with a previous history of TB compared to the ART-naive group (P < 0.0001 and P < 0.001, respectively). The most common treatment regimen of the subjects on antiretroviral therapy was d4T/3TC/EFV (37%). The median period on a d4T-containing regimen among the ART group was 13 months (IQR 8–20 months) and on an AZT-containing regimen was 12 months (IQR 7–18.5 months).

Measures of body composition showed significant differences between the groups, however, the sum of skinfolds was not different. Subjects in the ART group weighed significantly more (P = 0.0004), had a higher mean BMI (P < 0.01), greater mean waist-to-hip ratio (P < 0.0001) and greater median sagittal height (P < 0.0001).

### Comparison of Health related quality of Life Outcomes by ART status

The HRQoL outcomes did not differ between groups for self-care, usual activities, pain/discomfort and anxiety/depression (Table 2). The ART-naïve group, however, experienced a significantly greater problem with mobility than the ART group (9 vs 4%, P < 0.05). Multivariate analysis showed no significant differences between groups for self-care, usual activities, pain/discomfort and anxiety/depression (Table 3). In contrast, those in the ART-naive group were 3.08 times more likely to report problems with mobility than those in the ART group (95% CI 1.37 - 6.94). In addition, those obese were 2.78 times more likely to have increased mobility problems than those not obese (95% CI 1.24- 6.22).

**Table 2:** Health-related quality of life (EuroQol) of ART-naïve and ART groups

	**Total**	**ART-naïve**	**ART**	**P value**
	**N (%)**	**N (%)**	**N (%)**	
**Mobility**				
No problems	844 (93.5)	397 (91.3)	444 (95.5)	
Problems	59 (6.5)	38 (8.7)	21 (4.5)	0.010
**Self Care**				
No problems	897 (99.3)	430 (98.9)	467 (99.8)	0.084
Problems	6 (0.7)	5 (1.2)	1 (0.2)	
**Usual Activities**				
No problems	885 (98.2)	423 (97.5)	462 (98.7)	0.167
Problems	17 (1.9)	11 (2.5)	6 (1.3)	
**Pain/Discomfort**				
No problems	680 (75.3)	316 (72.6)	364 (77.8)	0.074
Problems	223 (24.7)	119 (27.3)	104 (22.2)	
**Depression/Anxiety**				
No problems	782 (86.6)	372 (85.5)	410 (87.6)	0.357
Problems	121 (13.4)	63 (14.5)	58 (12.4)	
**VAS score, median (IQR)**	80 (70–95)	80 (70–90)	90 (80–100)	<0.0001

**Table 3:** Multivariate analyses showing factors associated with the five Health related Quality of Life (EuroQol) domains

	**Mobility**		**Selfcare**		**Usual activities**		**Pain/discomfort**		**Anxiety/depression**	
**Variable**	**OR (95% CI)**	**p-value**	**OR (95% CI)**	**p-value**	**OR (95% CI)**	**p-value**	**OR (95% CI)**	**p-value**	**OR (95% CI)**	**p-value**
**ART status**										
ART-naïve vs ART	3.08 (1.63 - 7.89)	0.007	5.21 (0.45 - 60.27)	0.187	2.40 (0.66 - 8.75)	0.184	1.31 (0.88 - 1.95)	0.184	0.88 (0.53 - 1.89)	0.607
**CD4 count, cells/μL**										
> 200 vs ≤ 200	1.16 (0.55 - 2.47)	0.697	2.93 (0.28- 30.40)	0.367	0.75 (0.24 - 2.37)	0.622	0.81 (0.54 - 1.21)	0.311	1.14 (0.66 - 1.95)	0.646
**Age**	1.02 (0.99 - 1.06)	0.242	0.92 (0.80 - 1.06)	0.236	0.95 (0.89 - 1.03)	0.208	1.01 (0.99 - 1.03)	0.471	1.01 (0.99 - 1.04)	0.320
**Gender**										
Female vs male	0.44 (0.19 - 1.00)	0.050	1.90 (0.12 -30.97)	0.651	2.04 (0.39 - 10.77)	0.401	0.83 (0.52 - 1.31)	0.416	1.03 (0.55 - 1.91)	0.933
**Education**										
No education	1.00	1.00	1.00		1.00		1.00		1.00	
≤ Grade 12	0.37 (0.13 - 1.06)	0.065	0.028(0.002-0.309)	0.004	0.11 (0.02 - 0.58)	0.009	0.90 (0.41 - 1.94)	0.779	0.59 (0.23 - 1.51)	0.273
>Grade 12	0.41 (0.04 - 4.20)	0.450	-	-	0.31 (0.02 - 4.33)	0.382	1.14 (0.31 - 4.29)	0.841	0.59 (0.10 - 3.52)	0.565
**Employment**										
Unemployed	1.00		1.00		1.00		1.00		1.00	
Full or part time or other	0.71 [0.31 - 1.59]	0.400	0.75 (0.06 - 9.54)	0.826	0.31 (0.02 - 4.33)	0.458	1.34 (0.90 - 2.00)	0.150	0.59 (0.34 - 1.04)	0.067
**Previous TB**	1.54 (0.72 - 3.28)	0.267	2.23 (0.25 - 19.54)	0.469	3.10 (0.85 - 11.24)	0.086	1.12 (0.75 - 1.66)	0.583	0.51 (0.30 - 0.86)	0.012
**BMI category**										
Not obese	1.00		1.00		1.00		1.00		1.00	
Obese	2.78 (1.24- 6.22)	0.013	0.92 (0.07- 11.63)	0.947	1.09 (0.27 - 4.41)	0.906	1.20 (0.77 -1.86)	0.427	1.27 (0.74 - 2.17)	0.384

All differences were significant at P < 0.0.

The ART-naïve group had a median VAS score of 80 (IQR 70–90), which was lower than that of the ART group (median 90, IQR 80–100; P < 0.0001). Multivariate analyses showed a 5.61 point increases in VAS score in the ART group compared to ART-naïve group (95% CI 2.50 - 8.72; p < 0.001 (Table 4). Also, those with some source of income were 4.76 times more likely to have a higher EQ-5D VAS score than those unemployed (95% CI 1.63 -7.89).

**Table 4:** Multivariate analyses showing factors associated with Visual Analogue Scale score

**Variable**	**Coefficient**	**95% CI**	**p-value**
**ART status**			
ART vs ART-naïve	5.61	2.50 - 8.72	<0.001
**CD4 count, cells/μL**			
>200 vs ≤200	0.58	−2.63 - 3.79	0.724
**Age**	−0.034	−0.20 - 0.13	0.682
**Gender**			
Female vs male	0.66	−2.95 - 4.26	0.720
**Education**			
No education	1.00	1.00	
≤ Grade 12	0.21	−6.07 - 6.48	0.949
>Grade 12	−5.12	−15.33 - 5.09	0.325
**Employment**			
Unemployed	1.00	1.00	
Full or part time or other	4.76	1.63-7.89	0.003
**Housing density**			
<1.9	1.00	1.00	
≥ 2	1.76	−1.15 - 4.66	0.236
**Previous TB**	−2.09	−5.20 - 1.01	0.186
**BMI category**			
Not obese	1.00	1.00	
Obese	3.10	−0.38 - 6.57	0.081

Constant 82.04022, R-squared 0.0616, Root MSE 16.106.

All differences were significant at P < 0.05.

### Comparison of Health related quality of Life Outcomes by ART status according to CD4 count strata

When grouped according to CD4 count strata, there were no significant associations between ART-naïve and ART groups in most of the EQ-5D domains (Table 5). However, the ART-naïve group indicated having significantly greater problems under the CD4 count of >500 cells/μL in the anxiety/depression domain (22.4% vs 8.8%, p = 0.018) and significantly lower EQ-5D VAS scores under the CD4 counts of ≤200 cells/μL (median 80 (IQR 60–90) vs 90 (IQR 80–100), p = 0.0003) and 201–350 cells/μL (median 80 (IQR 70–90) vs 90 (80–100).

**Table 5:** Subjects reporting problems in the EQ-5D dimensions (%) and median EQ VAS score by ART status across CD4 count strata

	**Total**	**ART-naïve**	**ART**	**P value**
	**N (%)**	**N (%)**	**N (%)**	
**Mobility (CD4 count, cells/μL)**				
≤200	16 (6.9)	14 (8.6)	2 (2.9)	0.113
201-350	13 (5.4)	6 (6.0)	7 (5.0)	0.736
351-500	6 (3.9)	3 (4.7)	3 (3.3)	0.649
**>**500	16 (10.3)	10 (13.2)	6 (7.5)	0.244
**Self care**				
≤200	1 (0.4)	1(0.61)	0 (0.0)	0.511
201-350	1 (0.4)	1 (1.0)	0 (0.0)	0.236
351-500	1 (0.6)	0 (0.0)	1 (1.1)	0.403
**>**500	3 (1.9)	3 (4.0)	0 (0.0)	0.073
**Usual activities**				
≤200	6 (2.6)	6 (3.7)	0 (0.0)	0.104
201-350	4 (1.7)	2 (2.0)	2 (1,4)	0.733
351-500	2 (1.3)	0 (0.0)	2 (2.0)	0.235
**>**500	4 (2.6)	3 (4.0)	1 (1.3)	0.281
**Pain/discomfort**				
≤200	68 (29.2)	50 (30.7)	18 (25.7)	0.445
201-350	53 (22.1)	27 (27.0)	26 (18.6)	0.121
351-500	34 (21.8)	12 (18.8)	22 (23.9)	0.442
**>**500	42 (26.9)	22 (29.0)	20 (25.0)	0.578
**Depression/anxiety**				
≤200	32 (13.7)	22 (13.5)	10 (14.3)	0.873
201-350	33 (13.8)	10 (10.0)	23 (16.4)	0.154
351-500	18 (11.5)	7 (10.9)	11 (12.0)	0.845
**>**500	24 (15.4)	17 (22.4)	7 (8.8)	0.018
**VAS score, median (IQR)**				
≤200	80 (70–90)	80 (60–90)	90 (80–100)	0.0003
201-350	90 (70–95)	80 (70–90)	90 (80–100)	0.0004
351-500	90 (80–100)	82.5 (80–100)	90 (77.5-100)	0.455
**>**500	80 (70–90)	80 (70–90)	90 (80–100)	0.004

## Discussion

In this study that compared HRQoL outcomes between ART-naïve and patients on ART for 6 months or longer (who were mostly using a d4T-based first-line regimen) the key findings were: Subject’s EQ-VAS scores (representing the subject’s self-rated health state) improved significantly on ART; and when subjects were grouped according to their baseline CD4 counts, EQ-VAS scores were significantly lower in ART-naïve patients with CD4 counts ≤200 cells/μL and 201–350 cells/μL.

Our finding that study subject’s EQ-5D VAS scores improved significantly on ART, suggests that drug toxicities, especially those related to d4T have little impact on subects self-rated health. This study, therefore, support the findings of several studies [9-12] which report improved HRQoL outcomes on ART, despite participants being national first-line regimens containing d4T with known toxicities [14-25]. The study by Pitt and colleagues [9], for example, showed that drug toxicities, mainly those related to d4T, had little impact on mental health HRQoL scores during the first 48 weeks on ART.

We also found that, when subjects were grouped according to their baseline CD4 counts, EQ-VAS scores were significantly lower in ART-naïve patients with CD4 counts ≤200 cells/μL and 201–350 cells/μL. Our study findings corroborate recently reported studies. Mathews and colleagues found that EQ-5D VAS score improved with increasing CD4 cell count strata and were 65.4, 70 and 75 for CD4 counts <50, 50–199 and ≥200 cells/μL [41]. Anis and colleagues [42] showed that improvements in CD4 cell counts were associated with higher HRQoL scores (P = 0.08). Bhargava and colleagues [13] found the HRQoL of patients receiving ART increased significantly with improvement in CD4 count. Igumbor and colleagues [7] found weak but significant associations between CD4 cell counts and HRQoL in a cohort of treatment-naïve patients and those who had received ART for 12 months.

The strength of this study was the large sample size of HIV-infected individuals derived from a primary care clinic and it is one of the few studies that shed light on the impact of higher CD4 cell counts on HRQoL. However, the results of the study may only be generalizable to the study population and other similar settings. Our finding that the EQ-5D VAS was sensitive to ART use and ART eligibility (based on CD4 cell count) supports the use of this particular instrument in future assessments of HRQoL outcomes among HIV infected individuals in South Africa.

The study had the several limitations. Because this was a non-randomized study, the associations observed in the study are potentially biased due to residual confounding. We could not adjust for HIV-related symptom burden, duration on ART (among those on ART) and HIV-RNA viral load levels because all these factors known to influence HRQoL among HIV infected individuals [38] were not assessed in the present study. Furthermore, there may be limitations to the generalizability of study findings due to the CD4 count categories used in this study.

## Conclusions

In conclusion, the study data suggest that ART is effective in improving the HRQoL (that is, self-rated health state), including those subjects who were immunocompromised, which may be applicable to ART eligibility of the public sector ART program in South Africa. Further research is needed to examine the impact that long term ART use has on the HRQoL of HIV-infected people, as well as looking at long term government strategies to improve HIV-infected patient’s general health perception during the course of lifelong ART.

## Abbreviations

HIV: Human immunodeficiency virus; ART: Antiretroviral therapy; HRQoL: Health-related quality of life; EQ-5D: EuroQol five domains questionnaire; EQ-5D VAS: EuroQol visual analogue scale; d4T: Stavudine; 3TC: Lamuvidine; 3TC: Efavirenz; AZT: Zidovudine; NVP: Nevaripine.

## Competing interests

The authors declare that that they have no competing interests.

## Authors’ contributions

MDN, SJW, VEL designed the study. MDN with input from SJW and VEL analyzed the data. MDN and SJW wrote the paper with input from all the authors who each approved the final version.

## Pre-publication history

The pre-publication history for this paper can be accessed here:

http://www.biomedcentral.com/1471-2458/14/676/prepub
